# Rapid and comprehensive evaluation of microalgal fatty acids via untargeted gas chromatography and time‐of‐flight mass spectrometry

**DOI:** 10.1002/elsc.201900092

**Published:** 2019-09-16

**Authors:** Holger Morschett, Jochem Gätgens, Wolfgang Wiechert, Marco Oldiges

**Affiliations:** ^1^ Institute of Bio‐ and Geosciences IBG‐1: Biotechnology Forschungszentrum Jülich GmbH Jülich Germany; ^2^ Institute of Biotechnology RWTH Aachen University Aachen Germany

**Keywords:** *Chlorella vulgaris*, fatty acid, gas chromatography time‐of‐flight mass spectrometry, *in‐situ* transesterification, triacylglycerides

## Abstract

Due to their high triacylglyceride content, microalgae are intensively investigated for bio‐economy and food applications. However, lipid analysis is a laborious task incorporating extraction, transesterification and typically gas chromatographic measurement. Co‐elution induces a significant risk of fatty acid misidentification and thus, additional purification steps like thin layer chromatography are needed. Contrary to database matching approaches, solely targeted analysis is facilitated as compound identification is driven by matching retention times or indices with standard substances. In this context, a rapid workflow for the analysis of algal fatty acids is presented. *In‐situ* transesterification was used to simplify sample preparation and conditions were optimized towards fast processing. If results are needed at the very day of sampling, direct processing without a preceding drying step is feasible to obtain a rough estimate about the occurrence of the major compounds. Coupling gas chromatography and time‐of‐flight mass spectrometry enables untargeted analysis. Unknown compounds may be identified by structural reconstruction of their respective fragmentation patterns and by database matching to routinely avoid mismatches by co‐elution of disturbing agents. The developed workflow was successfully applied to derive the exact stereochemistry of all fatty acids from *Chlorella vulgaris* and a systematic shift depending on physiological state of the cells was confirmed.

**Abbreviations**
FA: fatty acid
FAME: fatty acid methyl ester

## 1 INTRODUCTION

Exploiting microalgal biomass, cost, and energy efficiency can be boosted via thorough utilization in a biorefinery concept 1. Depending on strain and process, fatty acids (FAs) and their esters may exceed 70% of cell dry weight 2. Besides membrane lipids 3, microalgae may contain significant amounts of stored lipids as triacylglycerides depending on the cultivation conditions 4. The composition of the lipid fraction is essential for its further use, e.g. the content of essential ω‐3 FAs is a key parameter for nutritional quality 5. On the contrary, for biodiesel application, such polyunsaturated FAs are unfavorable because the high degree of unsaturation impedes complying standards for biodiesel (e.g. European norm 14214) 6.

Methods for the analysis of FAs are well‐established for several decades. Mostly building on GC using a flame ionization detector, identification is driven by retention index matching with standards. The latter is clearly to be preferred with respect to general method robustness. Algal biomass is characterized by a high diversity of biomolecules 7 inevitably inducing risk of misidentification during chromatography due to co‐elution. Petkov and Garcia stressed this issue by pointing at several potential FA mismatches during GC analysis of *Chlorella* 8. Thus, sample preparation needs to integrate separation steps to minimize the fraction of potentially disturbing components. Thereby, these procedures become rather time and material consuming. Nowadays, standard protocols are mostly derived from extraction procedures established some decades ago 9, 10. Following these, biomass is first lyophilized, as thermal drying would result in oxidative degradation of FAs following lipid extraction with chloroform/methanol. The solvents are evaporated and the lipids transesterificated with acidic methanol. The resulting FA methyl esters (FAMEs) are recovered by hexane extraction and analyzed by GC. Preceding purification via thin layer chromatography to reduce co‐elution is strongly recommended 8. Nevertheless, such strategies can only reduce, but never exclude misidentifications.

Based on *Chlorella vulgaris* as model organism, a time efficient protocol enabling the unambiguous identification of microalgal FAs is presented. *In‐situ* transesterification and GC‐ToF‐MS are used as key technologies.

## 2 MATERIALS AND METHODS

### 2.1 Chemicals, strain, and media

All chemicals were obtained from Sigma‐Aldrich (Steinheim/Germany) and were of analytical or MS grade. *C. vulgaris* 211‐11b 11 (Culture Collection of Algae at the University of Göttingen, Germany) and the mineral medium reported in 12 were used for all experiments.

### 2.2 Cultivation conditions

150 mL medium in a 500 mL shake flask was inoculated from cryocultures to OD_750_ = 0.15. To obtain either exponentially growing or stationary cultures incubation time was set to 3 and 10 days, respectively. A tailored Multitron Pro shaking incubator (Infors HT, Einsbach/Germany) as specified in 13 was used to provide 200 µmol m^−2^ s^−1^, 2.5% (v/v) CO_2_, 25°C, 200 rpm and 25 mm shaking diameter.

**PRACTICAL APPLICATION**

There are different applications of microalgae biomass. In case of triacylglyceride production, a major focus is on the content of the fatty acids. However, triacylglyceride analysis is laborious incorporating extraction, transesterification and typically gas chromatographic measurement. Moreover, for nutritional application there can be demand for a specific fatty acid profile. This method allows rapid analysis and identification of the fatty acid profile. The developed workflow was successfully applied to derive the exact stereochemistry of all fatty acids from *Chlorella vulgaris* and a systematic shift depending on physiological state of the cells was confirmed.

### 2.3 Generation of FA extracts

Cultures were centrifuged for 10 min at 3939 g and 4°C (Labofuge 400R, Heraeus, Hanau/Germany), washed in 0.9% (w/v) NaCl and centrifuged again. The pellet was lyophilized (LT 105, Christ Gefriertrocknungsanlagen, Oserode am Harz/Germany), homogenized using a ceramic mortar and stored at −20°C prior to analysis.

Applying 1530 µL methanol and 170 µL H_2_SO_4_, 10 mg homogenized biomass wa*s in‐situ* transesterificated at 80°C, 1.5 h and 750 rpm (Thermomixer comfort, Eppendorf, Hamburg/Germany) unless otherwise specified. After cooling to room temperature, 300 µL desalted water and 1200 µL heptane were added and vigorously mixed for at least 1 min. The upper heptane phase was collected with a glass pipette and stored in glass vials at −20°C prior to analysis.

### 2.4 GC‐ToF‐MS operation

Analysis was carried out using a 6890 N gas chromatograph equipped with a 30 m EZ‐Guard VF 5 ms column and 10 m guard column (Agilent Technologies, Waldbronn/Germany) coupled to a Micromass GCT Premier time‐of‐flight mass spectrometer (Waters, Eschborn/Germany). Samples were injected by an MPS 2 autosampler (Gerstel, Mülheim an der Ruhr/Germany). Device operation is described in detail in 14.

A baseline noise subtracted fragment pattern was used to identify unknown compounds by comparison to the in‐house Jülich Polar Derivatives database as well as the commercial NIST11 and the freely available GMD 15 database. Unknown peaks were identified by structural combination of elemental compositions and verified by virtual fragmentation of the predicted structure. For additional verification, the analytical standards F.A.M.E. Mix RM 6, BAME Mix, Supleco® 37 Component FAME Mix (Sigma‐Aldrich, Steinheim/Germany), M 1603, M 1620 and M 1633 (Lipidox, Stockholm/Sweden) and *cis*‐7‐hexadecenoic acid methyl ester (Biomol, Hamburg/Germany) were used. Peak areas of individual FAMEs were normalized to the total peak area of all detected FAMEs in the respective sample. Relative fractions of co‐eluting compounds were derived from their corresponding extracted ion chromatogram signals.

### 2.5 Statistical analysis

For all statistical analyses, two‐sided t‐tests for unequal variances (95% significance level) were applied using Origin9.1.0G (OriginLab Corporation, Northampton/USA).

## 3 RESULTS AND DISCUSSION

### 3.1 Optimization of *in‐situ* transesterification

**Figure 1:**
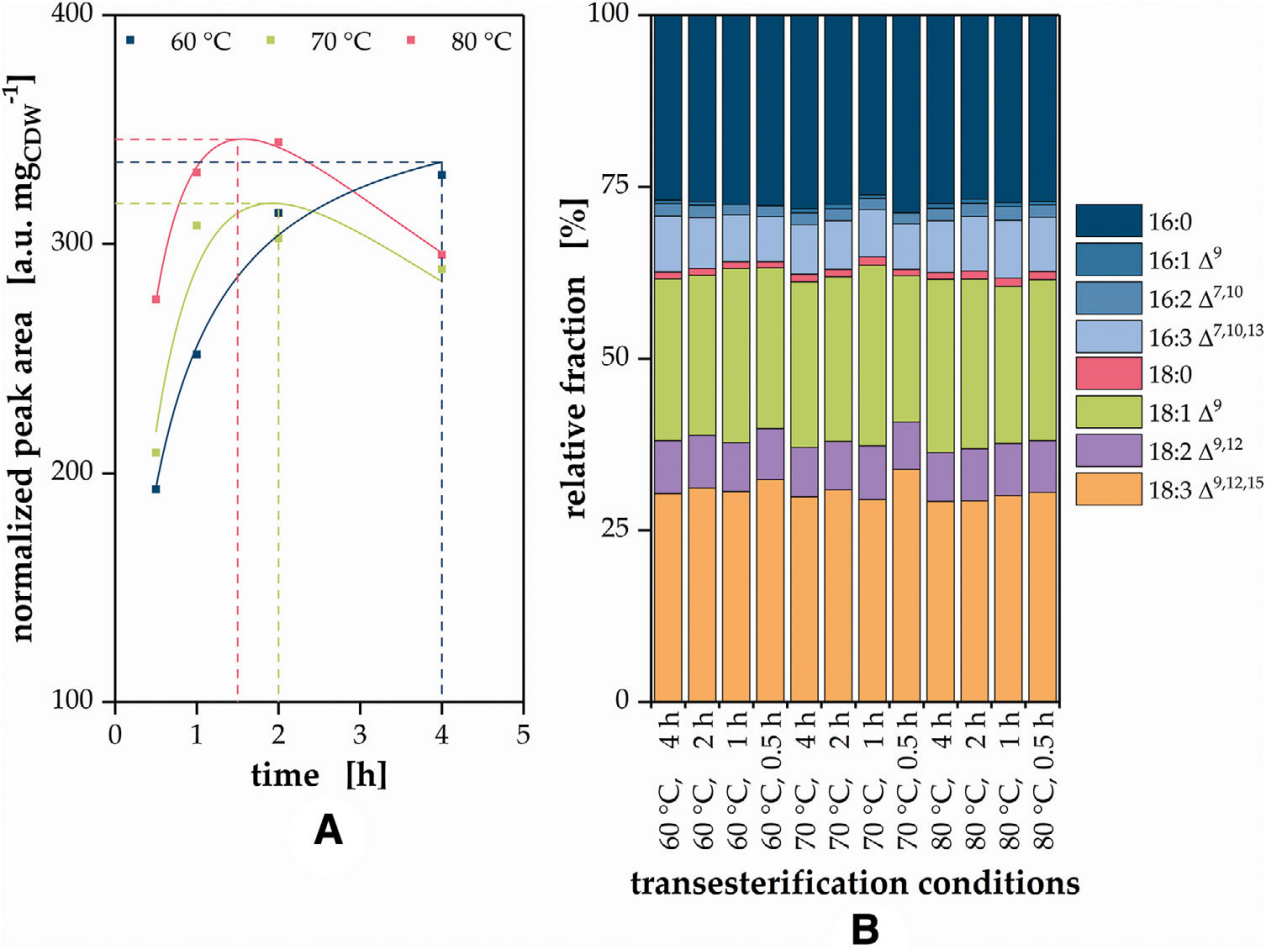
Optimization of the methanolic *in‐situ* transesterification. (A) The highest biomass‐specific signal intensity and thus sensitivity was achieved by 1.5 h *in‐situ* transesterification at 80°C. (B) No systematic influence of transesterification conditions on the resulting fingerprint could be observed

*In‐situ* lipid transesterification is a multistep process with the individual steps taking place simultaneously to a certain extend. The cells are disintegrated by disruption of cell wall and membrane accessing the cytosol containing the storage lipid bodies. The proton catalyzed transesterification of lipids with methanol releases FAMEs and free glycerol. To minimize processing times, the reaction course of *C. vulgaris* biomass *in‐situ* transesterification was investigated at different temperatures. In order to ensure comparable transesterification and extraction efficiency, applied amounts of biomass and reactants were kept constant along all experiments. The quantity of released FAMEs was monitored by the sum of all signal intensities from such FAs derivates detected by the MS normalized to the applied biomass (Figure 1A).

At 60°C reaction temperature, the biomass specific peak area steadily increased with incubation time from 192 a.u. mg_CDW_^−1^ (0.5 h) up to 330 a.u. mg_CDW_^−1^ after 4 h. Despite clearly showing saturation kinetics, it seemed that the maximum signal intensity was not reached yet and longer reaction times could further increase signal intensity. Higher temperatures resulted in an elevated initial value of 209 a.u. mg_CDW_^−1^ at 70°C and 276 a.u. mg_CDW_^−1^ at 80°C, respectively. Both conditions revealed an intermediate maximum after around 2 h at 318 a.u. mg_CDW_^−1^ (70°C) and after 1.5 h at 346 a.u. mg_CDW_^−1^ (80°C), subsequently dropping below 300 a.u. mg_CDW_^−1^ after 4 h.

The steady signal increase at 60°C indicated a limitation either by reaction rate or mass transfer and thus this temperature level proved unsuitable for fast sample analysis. On the contrary, higher temperatures resulted in higher biomass‐specific signal intensities and higher measurement sensitivity as the overall transesterification reaction proceeds faster with increasing temperature. The highest intensity was achieved at 80°C after 1.5 h. Consequently, these conditions were applied in further experiments. The signal intensity decrease after the specific local maxima applying 70°C and 80°C could be explained by thermal degradation processes, but was not further investigated in this context.

As depicted in Figure 1B (raw data provided in Supporting Information Table S1), only minor stochastic fluctuations, but no systemic influence of transesterification time and temperature were observed on the fingerprints. Although it might seem that single components only present in small amounts (e.g. 16:1 Δ^9^) might slightly increase over time, these changes are smaller than overall observable fluctuations. Thus, it is assumed that the investigated changes in reaction conditions seem to affect the present FAs to the same extend and thereby, the fingerprint is not distorted systematically.

Analysis can be further accelerated using wet instead of lyophilized biomass. However, time savings are at the price of biased fingerprints and especially trace components may drop below the sensitivity threshold (Supporting Information Table S2). Water from wet biomass acts as alternative nucleophilic agent reducing transesterification efficiency. Thus, such fast acquirable measures may only be used for rough estimation.

### 3.2 Comprehensive FA fingerprinting of *C. vulgaris*

As stressed in the “Introduction” section, the usability of algal biomass is strongly influenced by the composition of the lipid fraction. Despite some studies indicating differing species, there is strong evidence that the qualitative FA fingerprint of *Chlorella* is rather genus‐specific 8. Knowledge about the accurate stereochemistry of all involved FAs still remained incomplete, so far. The presented method was applied to set up a qualitative, but comprehensive fingerprint of *C. vulgaris* integrating the identification of all incorporated FAMEs by structural reconstruction of their respective fragments (Supporting Information Figures S3‐S12) as well as validation by analytical standards (Table 1).

**Table 1:** Comprehensive FA fingerprint from *C. vulgaris*. The exact stereochemistry of all FAs was identified upon structural reconstruction of molecule fragments from ToF‐MS and database matching. All double bonds are in Z configuration

Compound	Retention index [−]
14:0	1728
16:0	1929
16:1 Δ^7^	1906
16:1 Δ^9^	1910
16:2 Δ^7,10^	1899
16:3 Δ^7,10,13^	1904
18:0	2131
18:1 Δ^9^	2107
18:2 Δ^9,12^	2102
18:2 Δ^9,12,15^	2109

The qualitative spectrum assembled from comprehensive literature screening and experimental data by 8 could be fully confirmed using untargeted analysis. In addition, the exact stereochemistry of 16:2, 16:3, and 18:3 was identified to be 16:2 Δ^7,10^, 16:3 Δ^7,10,13^, and 18:3 Δ^9,12,15^. The highly sensitive measurements revealed that 16:1 may simultaneously occur in two constitutional isomers, namely 16:1 Δ^7^ and 16:1 Δ^9^.

Further FAs, for example < C_14_ and > C_18_, branched species or molecules with more than three double bonds were completely absent as would have been clearly identifiable by their characteristic fragment patterns. This renders the fingerprint of *Chlorella* rather simple compared to other microalgal species synthesizing a broader spectrum of FAs 16, 17, 18. Nevertheless, such compounds have already been claimed to contribute to *Chlorella*’s FA fingerprint 19, 20, but are likely attributable to taxonomic misclassification, contamination or the misidentification of FAs from GC data only 8.

**Figure 2:**
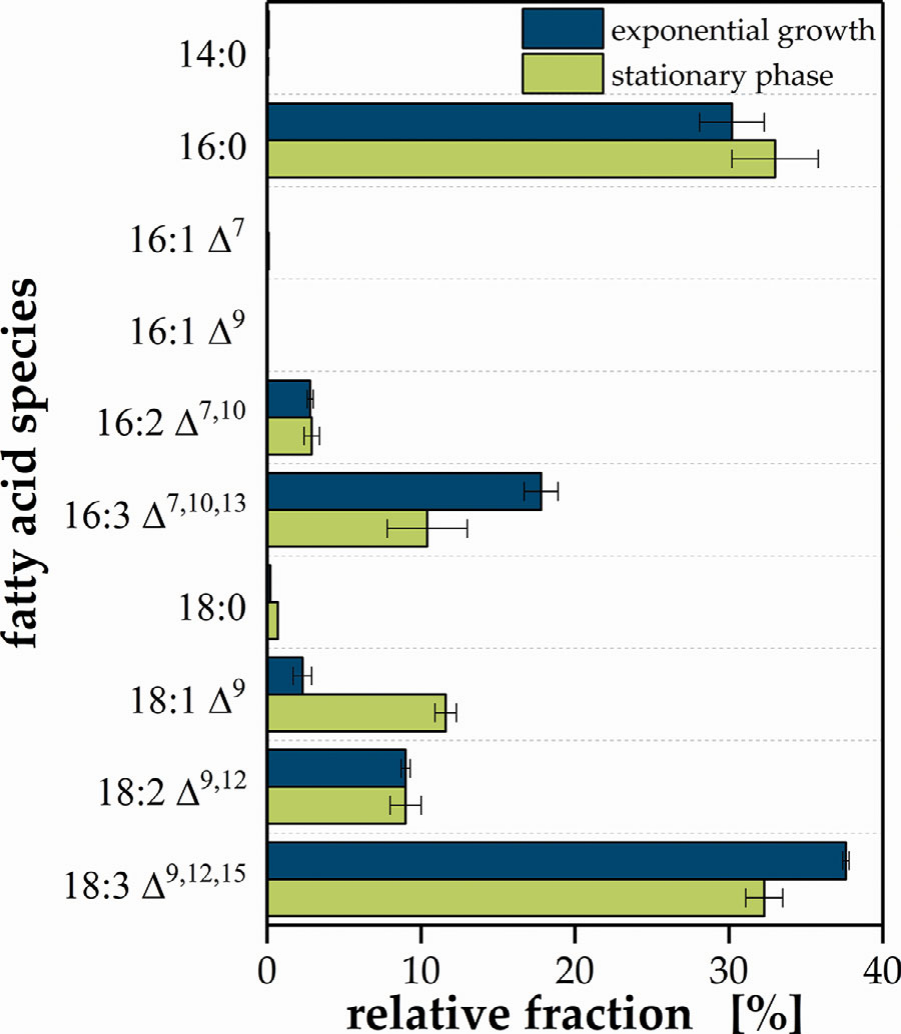
Relative FA fingerprint of exponentially growing and stationary *C. vulgaris* shake flask cultures. Error bars deviated from biological replicates (*n* = 3)

Besides the qualitative composition, literature reports about quantitative fingerprint variation depending on the cultivation conditions 18, 21. Typical cultivation processes start with an initial growth phase followed by growth‐decoupled production during which nutrient starvation represses growth and induces the synthesis of triacylglycerides and their intracellular accumulation in liposomes. To give proof for the applicability of the analytical method, cultivation phase induced changes in the FA fingerprint of *C. vulgaris* were monitored in shake flask experiments (Figure 2).

While 14:0, 18:0 and both stereoisomers of 16:1 occurred only in traces (< 1%) in both physiological states, 16:2 Δ^7,10^ and 18:2 Δ^9,12^ contributed approximately 3% and 9% to the total FAs in both states. In contrast, the major fraction consisted of 16:0, 16:3 Δ^7,10,13^, 18:1 Δ^9^ and 18:3 Δ^9,12,15^. Stearic acid with a share of approximately 30% showed only minor changes, whereas 18:1 Δ^9^ significantly increased from 2.3 ± 0.6% to 11.6 ± 0.7% during stationary phase. Simultaneously 16:3 Δ^7,10,13^ and 18:3 Δ^9,12,15^ shrank by ca. 42% and 14% from 17.8 ± 1.1% to 10.4 ± 2.6% and 37.6 ± 0.2% to 32.3 ± 1.2%, respectively. Altogether, the major fraction holds a share of ≈87% for both physiological states and was thus of highest importance for the total fingerprint.

These changes have significant impact on applicability of *Chlorella* biomass. The nutritional quality of *Chlorella* is not only determined by its protein content and composition 22 or the presence of so‐called nutraceuticals 23, but as well by its FA composition 24. Thus, the use of growing cells is preferable as they contain a higher share of essential ω‐3 FAs. On the contrary, these polyunsaturated species induce challenges when it comes to biofuel applications. A high share of such species leads to reduced oxidation stability and flow properties that fail biodiesel specifications like the European norm 14214 25, rendering FAMEs obtained from stationary cells favorable. In the case of an impermissible high share of polyunsaturated FAs costly catalytic hydrogenation or blending with fuels from alternatives sources is mandatory 6, 26, 27, 28.

## 4 CONCLUDING REMARKS

This study presents a rapid method for the analysis of microalgal FAs. To minimize sample processing times, *in‐situ* transesterification was used and FAMEs that can be analyzed by GC without further derivatization were obtained. Temperature increase to 80°C boosted transesterification velocity so that only 1.5 h incubation time was needed providing robust and reproducible results (7.6% average relative error). Analytical standards were used for database annotation in a first step. Based on that, FAMEs could be identified without batchwise use of standard substances by taking advantage of ToF‐MS analysis, database matching and structural reconstruction from fragmentation patterns. Co‐eluting impurities can be differentiated from FAMEs rendering further preparatory steps superfluous. The developed method was successfully used for the analysis of *C. vulgaris*' FA fingerprint while the exact stereochemistry of all FAs could be identified. Moreover, the method proved applicable to analyze cultivation condition dependent changes in FA composition.

## CONFLICT OF INTEREST

The authors have declared no conflict of interest.

## Supporting information

Supporting Information
