# Supplementary material for: Rapid and comprehensive evaluation of microalgal fatty acids via untargeted gas chromatography and time‐of‐flight mass spectrometry
Source: Eng Life Sci. 2019 Sep 16;19(12):1006–11. doi: 10.1002/elsc.201900092 (PMC6999052; doi:10.1002/elsc.201900092)
Supplement: Supplementary file 1 — Supporting Information [file ELSC-19-1006-s001.pdf]

Supporting Material

Rapid and comprehensive evaluation of microalgal fatty acids via untargeted GC-ToF-MS

Holger Morschett, Jochem Gätgens, Wolfgang Wiechert and Marco Oldiges

Table S1: Relative TAG fingerprint after *in-situ* transesterification of *Chlorella* biomass at different temperatures and reaction times.

		relative fraction [%]							
**temperature [°C]**	**time [h]**	**16:0**	**16:1 Δ^{9}**	**16:2 $\Delta^{7,10}$**	**16:3 $\Delta^{7,10,13}$**	**18:0**	**18:1 Δ^{9}**	**18:2 $\Delta^{9,12}$**	**18:3 $\Delta^{9,12,15}$**
60	0.5 h	27.8	0.2	1.6	6.5	0.9	23.4	7.4	32.4
	1 h	27.6	0.3	1.5	6.8	1.0	25.4	7.1	30.7
	2 h	27.2	0.5	1.8	7.4	1.0	23.3	7.7	31.2
	4 h	27.0	0.5	1.8	8.1	1.0	23.6	7.7	30.4
70	0.5 h	28.8	0.6	1.6	6.6	0.9	21.3	6.9	33.9
	1 h	26.2	0.7	1.6	6.9	1.2	26.3	7.8	29.5
	2 h	27.5	0.5	1.7	7.1	1.1	24.0	7.1	30.9
	4 h	28.2	0.3	1.7	7.2	1.1	24.1	7.2	29.9
80	0.5 h	27.1	0.5	1.8	7.9	1.2	23.5	7.6	30.5
	1 h	27.3	0.6	2.0	8.4	1.2	22.9	7.6	30.1
	2 h	26.8	0.6	1.9	7.9	1.2	24.7	7.6	29.3
	4 h	27.4	0.7	1.8	7.5	1.0	25.2	7.1	29.2

Table S2: Relative TAG fingerprint after *in-situ* transesterification of either wet or freeze-dried *Chlorella* biomass. Error bars deviated from biological replicates (n = 3).

	relative fraction [%]	
**compound**	**wet**	**freeze-dried**
14:0	0.0 ± 0.0	0.1 ± 0.1
16:0	35.4 ± 0.4	30.2 ± 1.2
16:1 Δ^{7}	0.0 ± 0.0	0.0 ± 0.0
16:1 Δ^{9}	0.0 ± 0.0	0.0 ± 0.0
16:2 $\Delta^{7,10}$	2.0 ± 0.0	2.8 ± 0.4
16:3 $\Delta^{7,10,13}$	14.7 ± 0.1	17.8 ± 1.3
18:0	0.2 ± 0.0	0.2 ± 0.0
18:1 Δ^{9}	1.3 ± 0.2	2.3 ± 0.1
18:2 $\Delta^{9,12}$	7.1 ± 0.2	9.0 ± 1.1
18:3 $\Delta^{9,12,15}$	39.4 ± 0.4	37.6 ± 1.8

Supporting Material

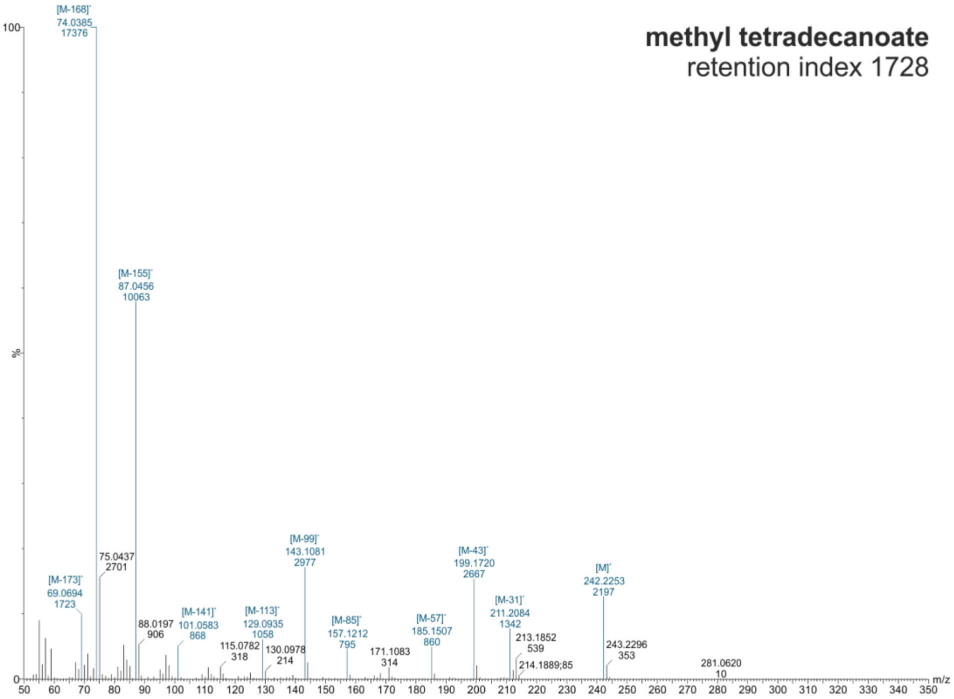

Figure S3: Fragmentation pattern of methyl tetradecanoate, 14:0, $C_{15}H_{30}O_2$.

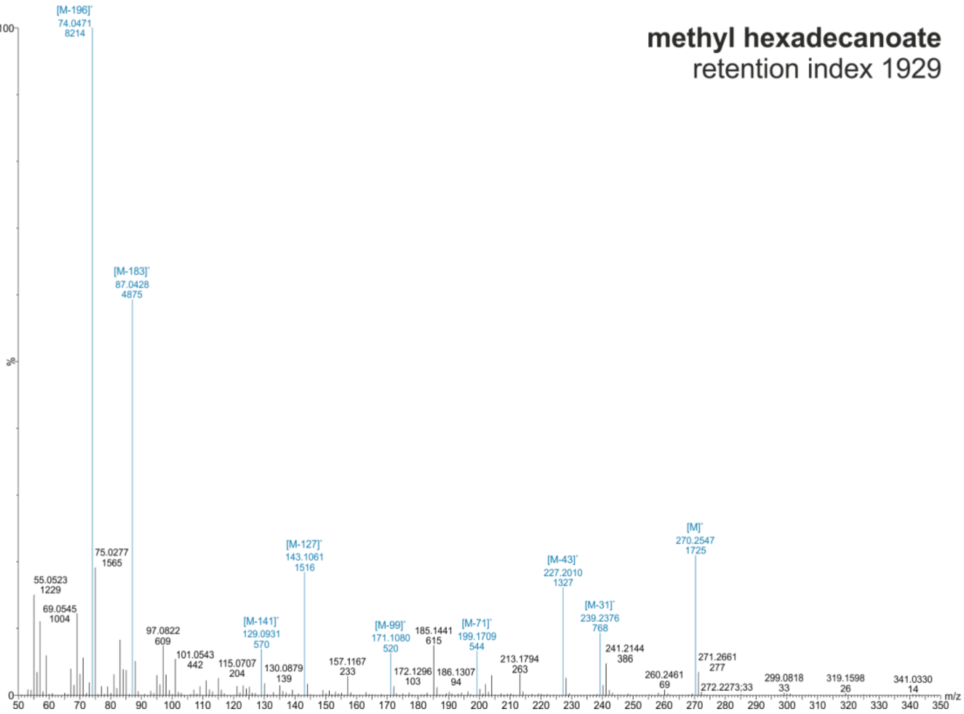

Figure S4: Fragmentation pattern of methyl palmitate, 16:0, $C_{17}H_{34}O_2$.

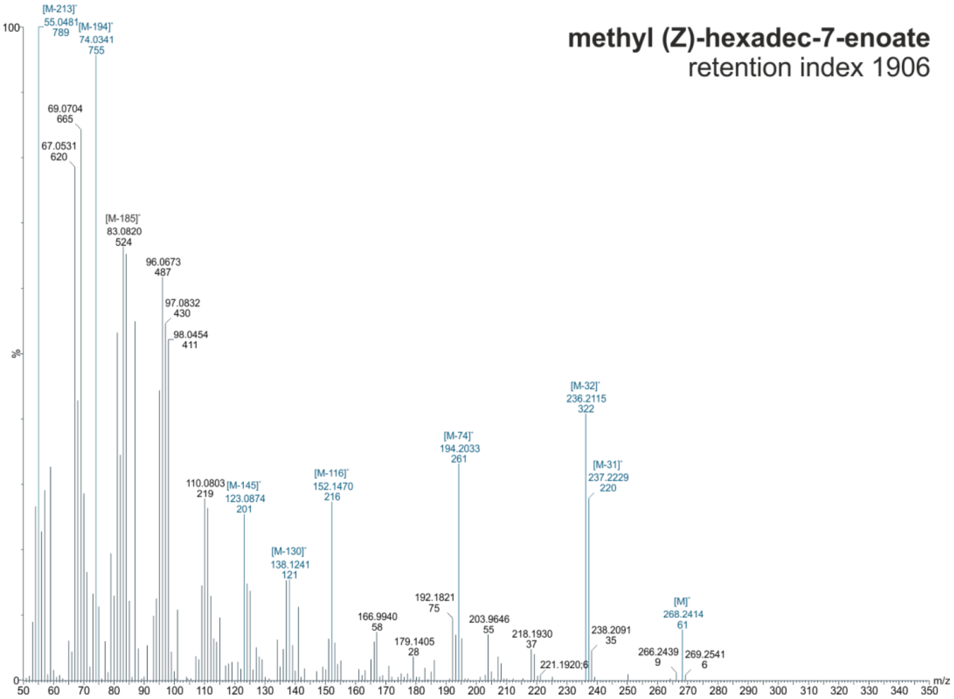

Figure S5: Fragmentation pattern of methyl (Z)-hexadec-7-enoate, 16:1 Δ^7, $C_{17}H_{32}O_2$.

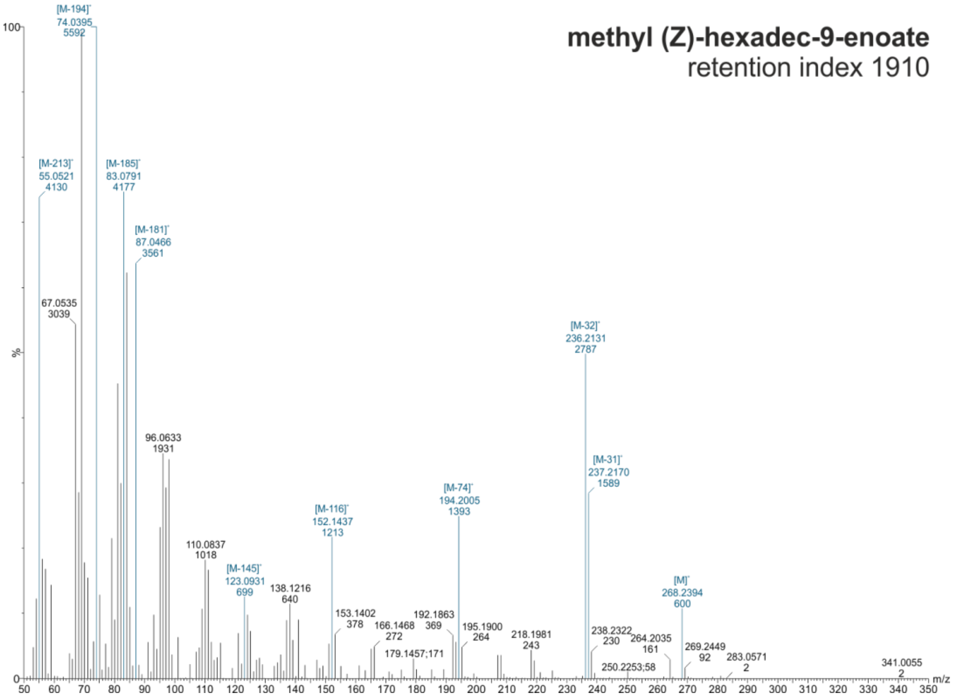

Figure S6: Fragmentation pattern of methyl (Z)-hexadec-9-enoate, 16:1 Δ^9, $C_{17}H_{32}O_2$.

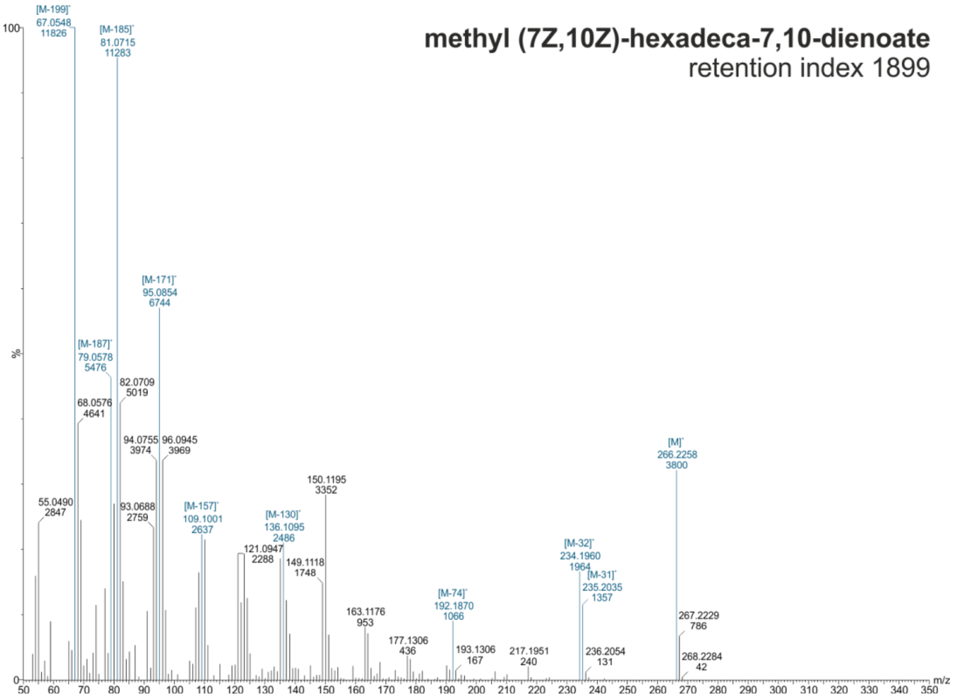

Figure S7: Fragmentation pattern of methyl (7Z,10Z)-hexadeca-7,10-dienoate, 16:2 $\Delta^{7,10}$, $C_{17}H_{30}O_2$.

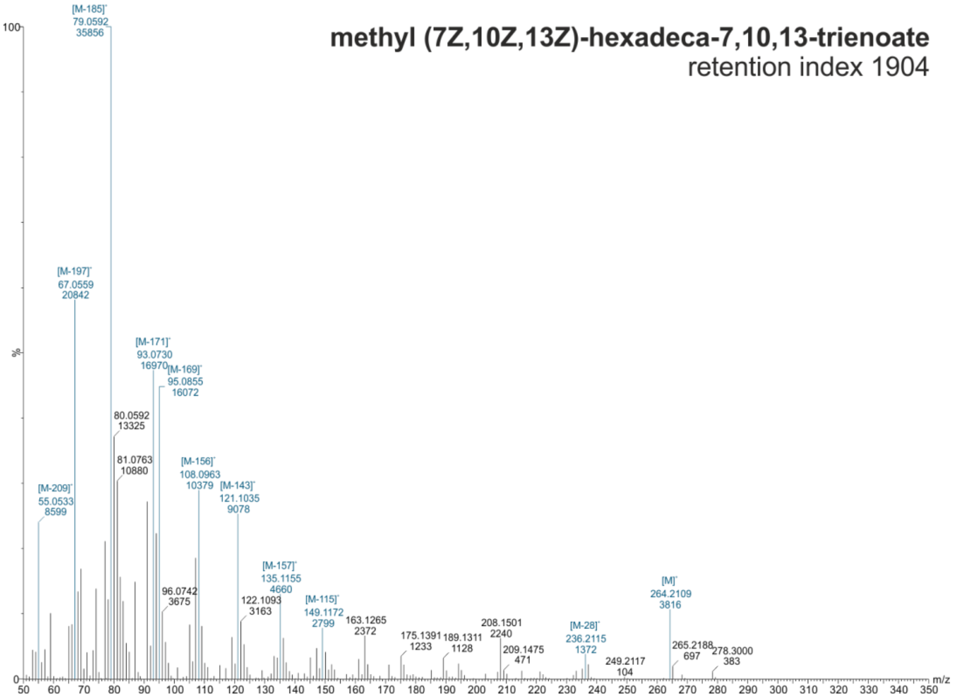

Figure S8: Fragmentation pattern of methyl (7Z,10Z,13Z)-hexadeca-7,10,13-dienoate, 16:3 $\Delta^{7,10,13}$, $C_{17}H_{28}O_2$.

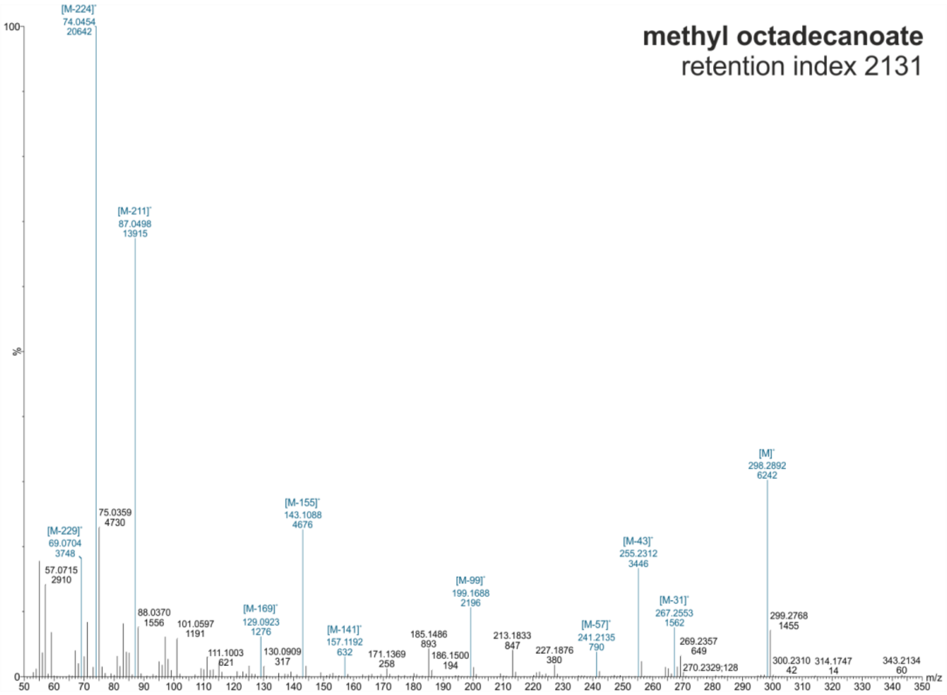

Figure S9: Fragmentation pattern of methyl octadecanoate, 18:0, $C_{19}H_{38}O_2$.

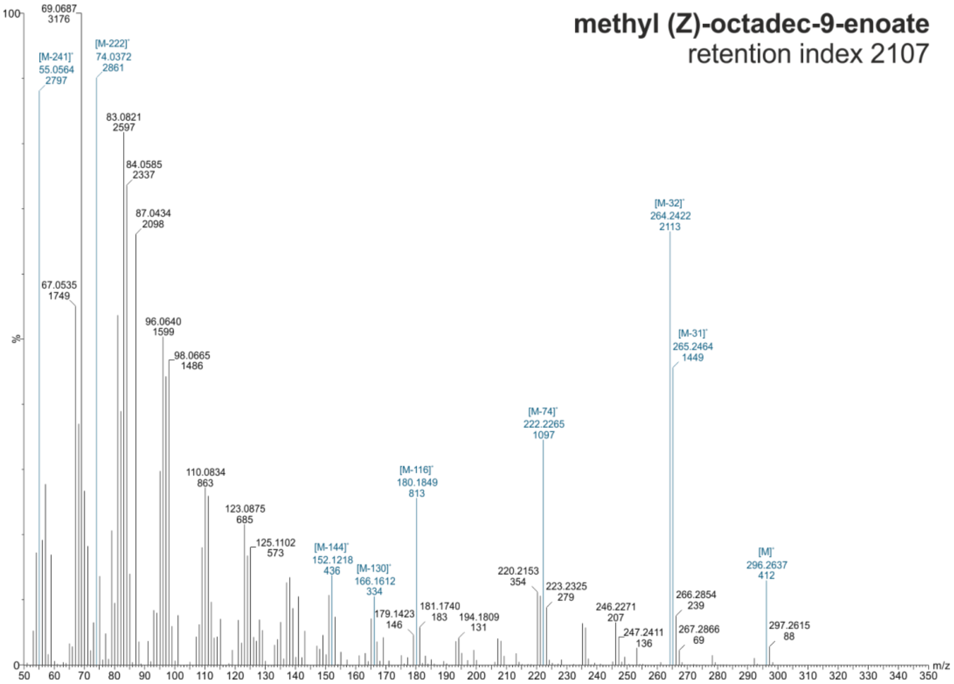

Figure S10: Fragmentation pattern of methyl (Z)-octadec-9-enoate, 18:1 Δ^9, $C_{19}H_{36}O_2$.

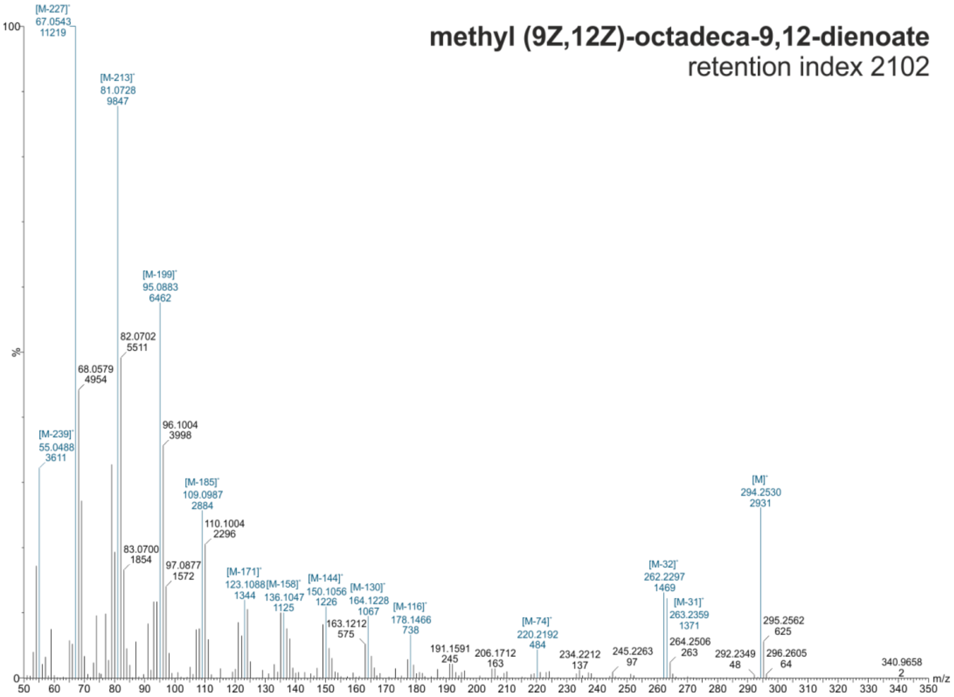

Figure S11: Fragmentation pattern of methyl (9Z,12Z)-octadeca-9,12-dienoate, 18:2 **Δ**^{9,12}, $C_{19}H_{34}O_2$.

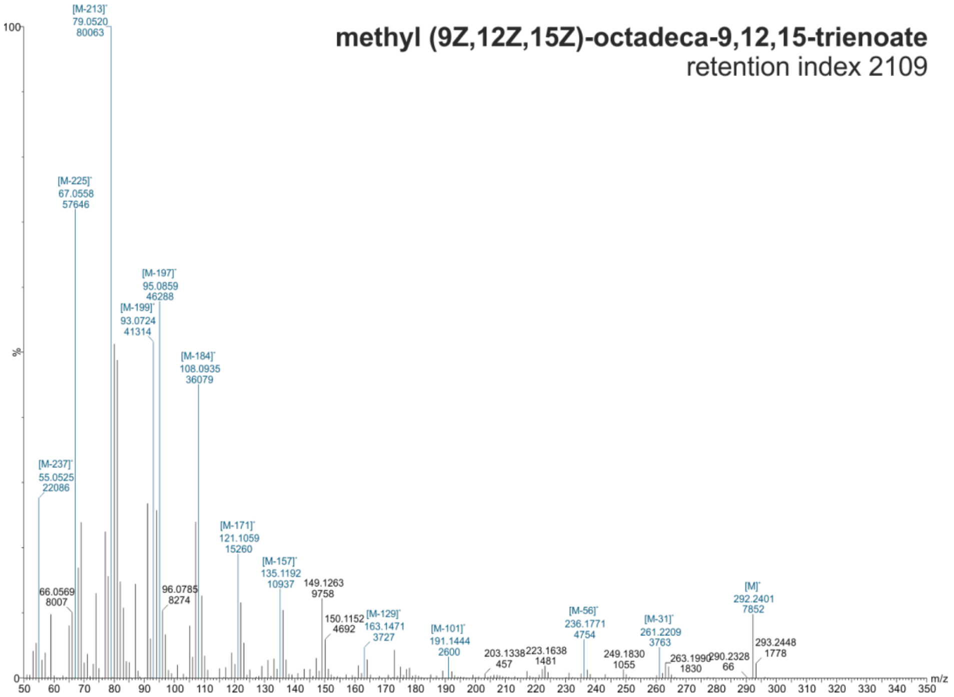

Figure S12: Fragmentation pattern of methyl (9Z,12Z,15Z)-octadeca-9,12,15-trienoate, 18:3 **Δ**^{9,12,15}, $C_{19}H_{32}O_2$.
